# Thyroid stimulating hormone (TSH): does it live up to its promises in the andrology laboratory?

**DOI:** 10.1186/s12610-026-00315-3

**Published:** 2026-07-07

**Authors:** Alireza Alizadeh Moghadam Masouleh, Monica Tobler, Mohammad Jafari Atrabi, Samira Vesali, Roya Hosseini, Joël Drevet, Andreas Gerd Schmutzler

**Affiliations:** 1 Department of Embryology, Reproductive Biomedicine Research Center, Royan Institute for Reproductive Biomedicine, ACECR, Tehran, Iran; 2 gyn-medicum, Center for Reproductive Medicine, Göttingen, Germany; 3 Institute of Pharmacology and Toxicology, University Medical Center Göttingen, Göttingen, Germany; 4 Department of Basic and Population Based Studies in NCD, Reproductive Epidemiology Research Center, Royan Institute, ACECR, Tehran, Iran; 5 Platform Degenerative Diseases, German Primate Center - Leibniz Institute for Primate Research, Göttingen, Germany; 6 German Center for Cardiovascular Research (DZHK), Partner Site Göttingen, Göttingen, Germany; 7 Department of Andrology, Reproductive Biomedicine Research Center, Royan Institute for Reproductive Biomedicine, ACECR, Tehran, Iran; 8 Faculty of Medicine, GReD Institute, EVALSEM, Université Clermont Auvergne, Clermont-Ferrand, France; 9 University Women’s Hospital, Christian-Albrechts-University, Kiel, Germany

**Keywords:** Thyroid-stimulating hormone (TSH), Sperm count, Alcohol, Smoking, Obesity, Hormone thyroïdienne stimulante (TSH), Nombre de Spermatozoïdes, Alcool, Tabagisme, Obésité

## Abstract

**Background:**

Research on Thyroid function in fertility/infertility has mainly focused on women, while men have scarcely been investigated. We investigated the association between TSH pattern and semen parameters, considering age, BMI, smoking and alcohol drinking. For 2021–2023, we examined all 346 male patients (infertile/fertile) in a Center for Reproductive Medicine at Germany. Age, BMI, smoking and detailed alcohol intake were recorded. A blood sample was taken from each patient in connection with the sperm collection to measure TSH, FSH, LH, prolactin, testosterone levels and the LH to testosterone ratio. Spearman’s correlation coefficients were used to determine correlations among hormones. Statistical analyses were performed using semen parameters as dichotomized using world health organization reference values.

**Results:**

The analysis explored relationships between age, BMI, smoking, alcohol consumption and semen parameters with TSH levels. The mean age and BMI were 35.8 ± 7.43 years and 27.1 ± 4.54 kgm^− 2^, respectively. Of all participants, 37% reported being smokers, alcohol consumption was 3% every day, 35% each week, 44% each month and 17% none. For TSH, we recorded 309 TSH levels, and the normal range was given as 0.4 to 4 mIU/L; 95% of the participants were in this category. Only a few participants were outside this range, with 1% below and 4% above. TSH levels were not found to be significantly affected by age, BMI, smoking, alcohol consumption or season. We found a positive correlation between TSH and prolactin (*r* = 0.199, *p* = 0.0001). Logistic regression showed that when TSH values were within the normal range, sperm counts were significantly higher (odds ratio:1.302, *P* = 0.043; confidence interval 95%:1.009–1.681).

**Conclusions:**

Male patients with abnormal TSH levels are not very common in this region (5%). Further studies regarding thyroid tests in andrology laboratory are required. TSH assessment in infertile couples should be routine in both partners, given the recorded worldwide increase in thyroid dysfunction.

## Résumé

**Contexte:**

La recherche sur la fonction thyroïdienne en fertilité/infertilité s’est principalement concentrée sur les femmes, tandis que les hommes ont été rarement étudiés. Nous avons étudié l’association entre le schéma de TSH et les paramètres du sperme, en tenant compte de l’âge, de l’IMC, du tabagisme et de la consommation d’alcool. Pendant la période 2021 – 2023, nous avons examiné tous les 346 patients masculins (infertiles/fertiles) dans un Centre de Médecine de la Reproduction en Allemagne. L’âge, l’IMC, le tabagisme et la consommation détaillée d’alcool ont été enregistrés. Un échantillon sanguin a été prélevé sur chaque patient en lien avec la collecte de sperme, afin de mesurer les hormones TSH, FSH, LH, prolactine, les niveaux de testostérone et le rapport LH/testostérone. Les coefficients de corrélation de Spearman ont été utilisés pour déterminer les corrélations entre hormones. Des analyses statistiques ont été réalisées en utilisant les paramètres du sperme dichotomisés à partir des valeurs de référence de l’Organisation Mondiale de la Santé.

**Résultats:**

L’analyse a exploré les relations entre l’âge, l’IMC, le tabagisme, la consommation d’alcool et les paramètres du sperme avec les niveaux de TSH. Les valeurs moyennes de l’âge et l’IMC étaient respectivement de 35,8 ± 7,4 ans et 27,1 ± 4,5 kgm-2. Parmi tous les participants, 37% ont déclaré fumer, la consommation d’alcool était de 3% chaque jour, 35% chaque semaine, 44% chaque mois et aucune chez 17%. Pour la TSH, nous avons enregistré 309 taux de TSH, et la plage normale était donnée entre 0,4 et 4 mIU/L ; 95% des participants appartenaient à cette catégorie. Seuls quelques participants étaient en dehors de cette fourchette, avec 1% en dessous et 4% au-dessus. Les niveaux de TSH n’étaient pas significativement affectés par l’âge, l’IMC, le tabagisme, la consommation d’alcool ou la saison. Nous avons trouvé une corrélation positive entre la TSH et la prolactine (r = 0,199, p = 0,0001).

**Conclusions:**

Les patients masculins présentant des taux anormaux de TSH sont peu fréquents dans cette région (5%). Des études supplémentaires concernant les tests thyroïdiens en laboratoire d’Andrologie sont nécessaires. L’évaluation de la TSH chez les couples infertiles devrait être courante chez les deux partenaires, compte tenu de l’augmentation mondiale des dysfonctionnements thyroïdiens.

## Introduction

Infertility is a worldwide problem, affecting over 100 million people [1]. It is estimated that the male factor contributes to 50% of these infertility cases, and this figure has been rising in recent years. Clinical semen analysis, which assesses a variety of parameters including sperm concentration and motility, a basic tool for the diagnosis and treatment of male infertility, regularly reports deterioration over the years [2–4]. Among the many genetic and environmental causes, endocrine and metabolic disorders, including hypogonadism, diabetes, obesity and adrenal dysfunction have been associated with male subfertility or infertility [5–8]. Beyond these conditions, it has been suggested that thyroid dysfunction may also affect male fertility [9], although this issue has been little studied and is somewhat controversial [10]. It is a fact that thyroid hormones (TH) receptors are expressed in the human testis where they modulate the functioning of Sertoli and Leydig cells [9]. The participation of TH in the physiology of testes before and during puberty is also well-known [11]. However, the thyroid role in adult testes and the extent to which it affects semen parameters and, consequently human male fertility, remains unclear [5, 12].

Thyroid dysfunction is fairly common in the general population, with a prevalence of approximately 1% for hyperthyroidism and 6% for hypothyroidism [13]. The progressive road for thyroid testing optimization is not detailed here, as it is well described by previous studies [14]. Routine monitoring of thyroid function relies primarily on the assessment of TSH (or thyrotropin), and it has been suggested that measurements of triiodothyronine (T3) and thyroxine (T4) could be recommended [14–16]. Fertility clinics and andrology labs worldwide mainly use the affordable TSH evaluation to assess thyroid function and consider thyroid function to be normal when TSH is in the range 0.4mIU/L < TSH<4.1mIU/L [17]. When TSH is outside this range, thyroid dysfunction, whether hyper-or hypothyroidism, is confirmed by T4 assessment.

A small number of reports have looked at semen parameters in situations of hyperthyroidism or hypothyroidism and compared them with control individuals [9–11, 13]. On the other hand, there is very little data exploring TSH levels in fertile cohorts compared to subfertile/infertile cohorts. As an example, Meeker et al., 2007 reported that TSH levels were not associated with semen parameters in a large cohort of US men [18]. Similarly, Nikoobakht et al., 2012 reported no association between serum TSH levels and seminal parameters in a smaller cohort of men diagnosed with hypothyroidism [19]. Similar conclusion of no association between serum TSH levels and semen parameters were issued by an Italian report [20] and a Greek one [11]. However, contradictory data can be found, with for example Lisovskaya et al., 2021 reporting a higher incidence of thyroid disorders in men from couples referred to infertility centers when compared to the general population [21]. Lotti et al., 2016 reported too that 7.4% of males from infertile couples did show subclinical hypothyroidism and 3.7% subclinical hyperthyroidism [20], percentages that are slightly above the frequency of thyroid disorders in the general population [22]. Moreover, subclinical hypothyroidism was associated with worse clinical outcome after IVF/ICSI, especially in men over 35 years of age [23]. In contradiction also with the reports finding no association between serum TSH level and male infertility was a Pakistani study showing that for each 1 mIU/L increase in serum TSH there was in parallel a 5.9 × 10^6^/ml decrease in sperm count and a 2.7% decrease in sperm motility [24]. Similarly, Venkateswara Rao et al., reported correlations between serum TSH level and sperm count as well as semen volume in Indian infertile men [25]. More recently, Bahreiny et al. in a meta-analysis study pointed out the adverse effects of subclinical hypothyroidism (SCH) on sperm quality parameters which shed more light on the importance of considering the thyroid function in andrology laboratories [26].

Based on these preliminary and conflicting reports it is generally assumed that evaluation of thyroid function is rather not pertinent in the assessment of the infertile male. Therefore, it is rarely evaluated and, as a logical consequence, thyroid dysfunction is rarely reported in the vast majority of oligo-astheno-teratozoospermic (OAT) men entering infertility clinics [27]. Only in men showing ejaculatory and erectile dysfunction is TSH evaluation recommended among others parameters [22, 28].

Kiel et al. reported both thyroid disorders and undiagnosed thyroid disorders are common in the adult German population, and undiagnosed thyroid disorders (morphological or functional) are found in up to 75% of the population. While there are data on hospital-based procedures such as surgeries, radioiodine treatment and thyroid scintiscans, little is known about guideline implementation and the prevalence of diagnostic procedures in ambulatory care [29]. This context prompted us to test via a retrospective study the association between TSH serum levels and semen parameters taking into account age, BMI, smoking, alcohol drinking and other classical sex hormone levels including FSH, LH, prolactin and testosterone. This was investigated using a rather large cohort of 346 men seen at the infertility clinic of Göttingen (Germany).

## Materials and methods

### Patients and study design

**Fig. 1:**
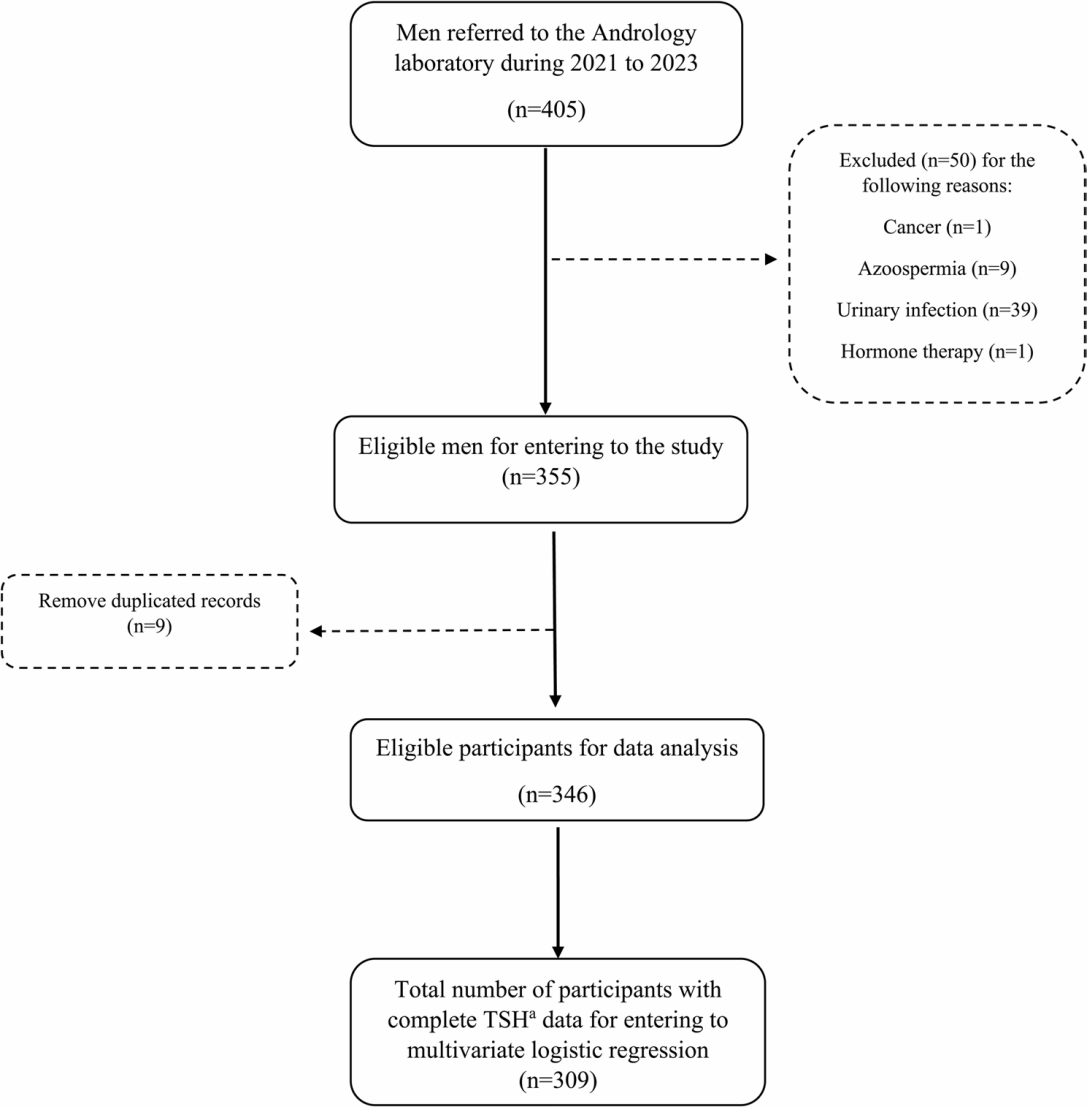
Patients’ recruitment flowchart. ^a^Thyroid-stimulating hormone

In a first screen, between 2021 and 2023, we examined all men who were referred for the first time to the andrology laboratory at gyn-medicum in Göttingen, Germany, for a semen analysis as part of a routine diagnostic procedure or pre-ART examination. Staff trained for the study measured weight and height, and body mass index (BMI) was calculated as the ratio of weight to height squared (in kilograms *per* square meter). All men completed a brief questionnaire at the time of recruitment by nursing staff and provided information on their health status: age, smoking habits (Yes or No), and alcohol drinking (none, rarely (once a month), occasionally (once a week) and regular (once a day)). Patient records were only included in the final analysis if they provided all the necessary information. Patients suffering from azoospermia, genital infections, undergoing hormone supplementation, or undergoing heavy medical treatments (radiotherapy and chemotherapy) were excluded. In accordance with these criteria, the final analysis included 346 fertile and infertile men (Fig. 1). The study was conducted in accordance with the Declaration of Helsinki as revised in 2013. This retrospective chart review study involving human participants was in accordance with the ethical standards of the institutional research committee. Ethical approval was received from the Ethics Committee of the Medical Faculty of the Christian-Albrechts-University, Kiel, Germany (D 479/24). Their appropriate processes have been followed. For the applicant on the one hand informed patient consent is waived under the German Law on Transplantation (TPG § 14), following compulsory European Regulations, thus submitting Clinical Fertility Centres under transplantation laws. On the other hand, it requires these Centres to analyse their own patient data continuously and anonymously by a compulsory quality control system.

### Semen analysis

The quality control program of the German Society of Andrology (QuaDeGA program, Münster, Germany) issues annual quality control certificates for our andrology laboratory (https://www.quadega.de/english/). At the clinic, semen samples were collected by masturbation after a period of sexual abstinence lasting 2 to 5 days, then stored in a sterile wide-mouthed, leak-proof container. Semen analysis included a macroscopic examination of appearance, viscosity, and volume was performed in accordance with World Health Organization’s guidelines (WHO, 2021). The samples were liquefied at 37 °C within 30 min. Semen volume was measured using conical graduated tubes, and spermatozoa were examined using a phase contrast microscope (Olympus Optical CH30RF200 Co., Ltd., Tokyo, Japan). Two expert embryologists assessed sperm concentration and motility from a 10 µL sample deposited on a clean counting slide (Cellvision 20-micron 10 × 10; CellVision, SB Heerhugowaard, Netherlands). Hemocytometers were allowed to rest for 10–15 min in a humid chamber to enable sedimentation of the suspended spermatozoa onto the counting grid before counting. The number of spermatozoa (×10^6^ sperm *per* ejaculate) was calculated by multiplying the sperm concentration by the semen volume. Motility assessments were performed by laboratory personnel using phase contrast microscope optics within 30–60 min after sample collection. Sperm motility was classified using a four-category scheme: rapid progressive, slow progressive, non-progressive, and immotile. At least 200 spermatozoa were assessed in each replicate motility count.

### Blood sampling and analysis method

A single, non-fasting, blood sample was collected by venipuncture from each participant on the same day as the semen sample was collected. TSH, FSH, LH, prolactin, and testosterone levels were measured by the Amedes Laboratory (Amedes Medizinische Dienstleistungs GmbH, Göttingen, Germany) using an electrochemiluminescence immunoassay (ECLIA) kit via the E801 module, Cobas8000 system (Roche Diagnostics GmbH, Mannheim, Germany). Intra-assay and inter-assay coefficient of variation (CV) were given for: TSH (1.3 and 3.2%), FSH (1.5 and 3.5%), LH (1.2 and 1.5%), prolactin (2.2 and 3.2%) and testosterone (2.3 and 3.9%), respectively.

### Statistical analysis

Data analysis was performed using SPSS software version 24.0 (IBM Corp., Armonk, NY, USA). Values are expressed as means ± standard deviation (SD) or percentages. Pearson’s correlation coefficients were used to determine correlations between hormones. Statistical analyses were performed on semen parameters, dichotomized according to WHO reference values. Multivariate logistic regression analysis was used to explore the relationship between age, BMI, smoking, alcohol consumption, and semen parameters (dichotomized based on the WHO reference thresholds). Men with values above the reference values for the three seminal parameters analyzed were used as comparison subjects in the multivariate logistic regression. The level of statistical significance was considered as *p* < 0.05.

## Results

### The demographic characteristics

The demographic characteristics and sex hormones measurements of the participants are presented in Table 1. The mean age of participants was 35.8 years, and the mean BMI was 27.2 kg/m2. Of the 346 men recruited for the study, the distribution of the cohort in terms of body weight was as follows: 37% were considered normal weight, 37% were overweight and 26% were obese. The study included more non-smokers (63%) than smokers (37%), and most participants reported drinking alcohol rarely (44%) or occasionally (35%). Among all participants, 3% reported drinking alcohol regularly and 17% did not drink alcohol.

**Table 1:** Demographic characteristics and hormone levels of the participants^a^

Variable		Value
Age (year) mean ± SD^b^		35.79 ± 7.44
BMI^c^ (kg/m^2^) mean ± SD		27.18 ± 4.55
BMI groups n (%)	Normal (≤ 24.9)	126 (36.6%)
	Overweight (25 to ≤ 29.9)	129 (37.5%)
	Obese (≥ 30)	89 (25.9%)
Smoking n (%)	Yes (≥5 cigarettes/day)	127 (37.2%)
	No	214 (62.8%)
Alcohol consumption n (%)	No	58 (17.2%)
	Rarely (Each month)	150 (44.4%)
	Occasionally (Each week)	119 (35.2%)
	Regular (Every day)	11 (3.3%)
FSH (IU/L) mean ± SD		5.25 ± 4.42
median (IQR)^d^		4.15 (2.8–6.15)
Normal Range (IU/L)		1.5–12.4
LH (IU/L) mean ± SD		5.08 ± 2.350
median (IQR)		4.55 (3.38–6.42)
Normal Range (IU/L)		1.24–7.8
Prolactin (mIU/L) mean ± SD		307.06 ± 152.78
median (IQR)		276.5(194–384.25)
Normal Range (mIU /L)		53–360
Testosterone (µg/L) mean ± SD		4.32 ± 1.76
median (IQR)		4.15 (3.01–5.33)
Normal Range (µg/L)		3.09–10.81
LH: Testosterone ratio, mean ± SD		1.43 ± 0.08
median (IQR)		1.16 (0.76–1.55)
Thyroid-stimulating hormone (TSH) (mlU/L) mean ± SD		1.97 ± 1.06
median (IQR)		1.76 (1.27–2.41)
Normal Range (mlU/L)		0.4–4

^a^The data were presented in descriptive form (mean ± standard deviation or frequency/percentage)

^b^Standard deviation

^c^*BMI* body mass index

^d^*IQR* interquartile range

### Hormonal status and semen parameters

The participants’ sex hormone levels did not follow a normal distribution, so these variables were presented as median and interquartile range (IQR). Hormone levels were all within normal reference ranges as defined by WHO (Table 1). For TSH, we recorded 309 TSH levels and with the normal range being 0.4 mIU/L < TSH<4 mIU/L, 95% of participants fell within this category. Only a few participants were outside this range, with 1% below and 4% above. In addition, two subjects had clearly low TSH levels (= 0.01 mUI/l) and one subject had a clearly high TSH level (= 8.6 mUI/l).

Table 2 shows the distribution of classic semen parameters, including sperm concentration, count, and motility (total and progressive) in the cohort analyzed. Table 3 shows that among the various sex hormones evaluated, only one significant positive correlation was found between TSH and Prolactin (*r* = 0.199, *p* = 0.0001), while a tendency toward a negative correlation was suggested between TSH and LH (*r*=-0.101, *p* = 0.073). Using multivariate logistic regression analysis (Table 4), semen parameters (dichotomized based on WHO reference cut-offs) were regressed on TSH levels, taking into account age, BMI, smoking, alcohol consumption and season, although these factors are known to be potential confounders influencing TSH levels. Unsurprisingly, age was found to be a factor influencing sperm count, concentration, and total and progressive motility. The analysis also showed that when TSH values were within the normal range, sperm count was significantly higher. Among the few participants with above-normal TSH values, the frequency of abnormal sperm parameters (count, total and progressive motility) was higher.

**Table 2:** Distribution of semen parameters for male subjects^a^

	Mean	Std. Deviation	Median (IQR)^b^	Min.	Max.	Range
Volume (ml)	3.22	1.65	3 (2–4)	0.50	9.5	9
Sperm Concentration (×10^6^/ml)	54.54	40.07	47 (22–83)	0.55	223.00	222.45
Sperm Count (×10^6^ sperm per ejaculate)	167.14	143.61	126 (62–248)	0.99	892.80	891.81
Total Motility (%)	45.24	15.02	48(37–55)	0.00	80.00	80
Progressive motility (%)	34.48	13.71	36 (26–43)	0.00	69.00	69

^a^Data was shown as descriptive (mean ± SD or median)

^b^*IQR* Interquartile range

**Table 3:** Relationship between sex hormones level and Thyroid-stimulating hormone^a^

	Thyroid-stimulating hormone	
	*r*	Sig. (2-tailed)
FSH (IU/L)	-0.013	0.823
LH (IU/L)	-0.101	0.073
Prolactin (mIU/L)	**0.199**	**0.0001**
Testosterone (µg/L)	-0.093	0.102
LH: Testosterone ratio	-0.031	0.588

^a^Statistical significance using the Pearson correlation coefficient

**Table 4:** Association of TSH with sperm parameters using logistic regression^a^

		Volume (ml)					Sperm Concentration (×106/ml)					Sperm Count(×106 sperm per ejaculate)					Total Motility (%)					Progressive motility (%)				
		B^b^	Sig.	OR^c^	95% CI^d^ for OR		B	Sig.	OR	95% CI for OR		B	Sig.	OR	95% CI for OR		B	Sig.	OR	95% CI for OR		B	Sig.	OR	95% CI for OR	
					Lower	Upper				Lower	Upper				Lower	Upper				Lower	Upper				Lower	Upper
Age		0.001	0.96	1.00	0.95	1.06	0.05	**0.007**	1.05	1.01	1.10	0.05	**0.01**	1.05	1.01	1.1	0.06	**0.0001**	1.06	1.03	1.10	0.06	**0.0001**	1.06	1.03	1.10
BMI^e^		0.029	0.48	1.02	0.95	1.11	0.004	0.90	1.004	0.93	1.07	0.01	0.63	1.01	0.95	1.08	-0.04	0.15	0.95	0.90	1.01	-0.03	0.30	0.96	0.91	1.02
Smoking (yes)		-0.24	0.55	0.78	0.35	1.74	-0.06	0.05	0.05	0.28	1.00	-0.56	0.08	0.56	0.29	1.07	0.10	0.69	1.11	0.65	1.88	0.14	0.60	1.15	0.66	2.0
Alcohol intake	Rarely	12.65	0.99	19.31	0.126	24.49	1.40	0.21	4.06	0.44	36.86	1.42	0.20	0.41	0.45	38.1	1.08	0.21	2.95	0.54	16.11	0.71	0.41	2.03	0.36	11.26
	Occasionally	12.39	0.99	18.97	0.365	17.41	1.02	0.34	2.78	0.32	23.74	0.99	0.36	2.70	0.31	23.31	0.66	0.42	1.93	0.38	9.85	0.42	0.60	1.53	0.30	7.82
	Regular	12.4	0.99	19.20	0.536	21.86	0.55	0.61	1.74	0.20	15.16	0.42	0.70	1.52	0.17	13.54	0.82	0.32	2.28	0.44	11.68	0.69	0.40	1.99	0.39	10.22
Season	Summer	-0.37	0.49	0.68	0.23	2.00	-0.14	0.74	0.86	0.35	2.07	0.28	0.52	1.33	0.54	3.27	0.30	0.39	1.36	0.67	2.75	0.34	0.34	1.41	0.68	2.92
	Fall	-0.38	0.49	0.67	0.22	2.07	0.41	0.33	1.51	0.64	3.53	0.55	0.22	1.74	0.71	4.28	0.39	0.28	1.48	0.71	3.07	0.14	0.71	1.15	0.53	2.47
	Winter	-0.17	0.97	0.98	0.35	2.73	-0.03	0.93	0.96	0.40	2.32	0.27	0.56	1.31	0.52	3.26	0.35	0.33	1.42	0.69	2.90	0.34	0.36	1.40	0.67	2.93
THS^f^		-0.05	0.78	0.95	0.65	1.37	0.09	0.50	1.09	0.83	1.43	0.26	**0.04**	1.30	1.01	1.68	0.03	0.79	1.03	0.82	1.29	-0.01	0.91	0.98	0.77	1.25

^a^Statistical significance using the logistic regression. Model adjusted for Age, TSH, BMI, Smoking (no smoking as incept), Alcohol intake (no consumption as incept), Season (spring as incept). ^b^*B* Beta coefficient, ^c^*OR* Odds ratio, ^d^*CI* Confidence interval, ^e^*BMI* Body mass index, ^f^*TSH* Thyroid-stimulating hormone

## Discussion

Worldwide, clinical practice assisted reproductive technology (ART) guidelines do not recommend routine screening for asymptomatic thyroid dysfunction [30]. However, the rates of thyroid function tests and diagnostic procedures have increased in recent years in many countries [14, 31, 32]. A study conducted in Germany found that the age-standardized prevalence (*per* 1,000 persons) of at least one TSH measurement increased between 2008 and 2017, from 165 to 238 in men [33]. Our study is a novel approach to examine the link between TSH levels and sperm parameters in a fertility clinic in Germany considering life style. As this was a retrospective study, we were unable to establish a causal relationship.

In our study, the data revealed that in a small sample size of 309 men who underwent TSH testing, 95% of participants were within the normal TSH range. Only a few participants were outside this range, with 1% below and 4% above. Along with the study by Trummer et al. in 2001 [34], the present report is one of the few studies providing detailed information on TSH levels and sperm quality in Germany. It sheds new light on the relationship between normal TSH levels and sperm quality parameters in an andrology laboratory at a European fertility clinic, taking into account age, BMI, smoking, and alcohol consumption.

In our study, sperm counts were significantly higher when TSH values were within the normal range. In addition, sperm concentration and motility were also found to be increased in men with normal TSH levels, although this was not statistically significant but clinically relevant. Unlike in western countries, several studies conducted in Asia have examined thyroid hormone levels in men referred to fertility clinics and, recently, Zhang et al. confirmed our findings in China [35]. Their retrospective study with 8478 men who consulted for fertility problems, revealed that in non-azoospermic men, high levels of free T3 was associated with better sperm motility, and that only a weak link was observed between high levels of free T4 and increased sperm concentration. Their findings suggest that Thyroid hormone levels are associated with measurable changes in sperm quality, with motility characteristics being particularly affected [35]. Contrary to our findings, a study conducted by Venkateswara Rao et al., in 2022, in the form of a prospective case-control survey of 100 infertile men and healthy controls of the same age in India, revealed a significant inverse correlation between TSH levels and semen volume, sperm motility, and sperm morphology [25]. They mentioned that the correlation between TSH and sperm parameters had been calculated for the entire male population composed of case groups and healthy control groups, given the absence of a statistically significant difference between the two groups with regard to TSH, and the statistically significant difference between the two groups with regard to sperm parameters. Similarly, Rehman et al. in 2020 conducted a study in Pakistan involving 376 men at the age of 25 to 55 years. Their results show that an increase in TSH levels led to a significant decrease in three sperm parameters, namely sperm count, motility and normal morphology [24]. They sought to establish a correlation between TSH and sperm parameters. A moderate negative correlation was observed between TSH and sperm count and motility. In our study, multivariate linear regression of sperm count and thyroid hormone profile confirmed that TSH was one the most influential factor on sperm count.

In worldwide population-based studies, the prevalence of thyroid disorders (TD) has been estimated at between 2% and 6% [13, 36]. Among European and American adults TD is even higher picking at 6.6% [37]. Aggregated data on drug dispensing in Germany showed a marked increase in the prescription of thyroid hormones between 2006 and 2016. In 2021, levothyroxine was among the five most frequently prescribed drugs in Germany [33]. According to our findings, among the few participants with above-normal TSH values (TSH > 4 mIU/L), the frequency of abnormal sperm parameters (count, motility, and progression) was higher.

In agreement with Rehman et al. [24], who highlighted a higher frequency of SCH in the group with altered sperm parameters, we suggest the need for screening for TD in couples suffering from infertility which confirmed with more recent review [38] and meta-analysis [26] studies. On the contrary, a retrospective study conducted on a population of infertile men with or without SCH found no link between mild thyroid dysfunction and sperm quality [23]. Another study concluded that hypothyroidism had the most significant impact on sperm concentration, motility and morphology [19]. However, numerous other studies have not corroborated this conclusion and have led the authors to advise against routine screening of thyroid function in infertile couples. For example, a study conducted by Lotti‘s team in Italy found no positive correlation between TSH, FT3 and FT4 and sperm parameters (total sperm count, motility and morphology) [20]. Similarly, Trummer et al. in Germany found no effect of increased or decreased TSH, FT4 and FT3 values on sperm analysis [34].

The lack of consistency between TSH level and sperm parameters in men can be explained by confounding factors [14] known to influence thyroid function, including age [39], BMI [40], smoking [41], and alcohol consumption [42]. Therefore, in our results, the above confounding variables were included in a logistic regression to determine the pure association between TSH and sperm parameters. In most previous similar studies, only two confounding factors, age and BMI, were taken into account in the relationship between TSH and sperm parameters. Here, we also included smoking and alcohol consumption. None of the above known confounding factors appeared to influence the relationship between TSH and sperm parameters.

Our findings about TSH levels in andrology laboratory, suggest further studies are warranted in order to confirm the necessity for thyroid tests in infertile couples [43]. Ultimately, in order to generalize our research, the crucial role of iodine and the complex relationship between iodine and selenium levels (which greatly depend on geographical location) in thyroid function should be taken into account in future studies.

*Limitations of the Study*. This study has some limitations that should be considered when interpreting the results. First, its retrospective and single-center design may limit the generalizability of the findings. Second, the number of patients with abnormal TSH levels was relatively small, which reduces the statistical power of subgroup analyses. In addition, thyroid function was assessed using TSH alone, as T3 and T4 measurements were not consistently available; this prevented us from distinguishing between subclinical and overt thyroid dysfunction. Sperm morphology was not included due to incomplete and non-standardized data during the study period, which may have limited the overall assessment of semen quality. Although sperm concentration and motility were evaluated by trained personnel with good agreement, interobserver variability was not formally quantified in detail. Finally, some of the observed differences did not reach statistical significance, and given the sample size, these results should be interpreted with caution. Larger prospective studies, including a full thyroid profile and standardized semen analysis, are needed to confirm these findings and better clarify the role of thyroid function in male fertility.

## Conclusion

In conclusion, male patients with abnormal TSH levels are not very common in this region of Germany. A normal TSH level appears to be associated with a higher sperm count. Further studies regarding thyroid tests in andrology laboratory are required. It might be useful to routinely measure TSH levels in both partners of infertile couples, given the global increase in thyroid disorders and iodine deficiency.

## Data Availability

No datasets were generated or analysed during the current study. The data that support the findings of this study are available from the corresponding authors upon reasonable request.

## Abbreviations

ART: Assisted reproductive technology
BMI: Body mass index
ECLIA: Electrochemiluminescence immunoassay
IQR: Interquartile range
OAT: Oligo-astheno-teratozoospermic
T: Testosterone
T3: Triiodothyronine
T4: Thyroxine
TD: Thyroid disorders
TH: Thyroid hormones
TSH: Thyroid stimulating hormone
WHO: World health organization
